# Editorial

**Published:** 1838-10-24

**Authors:** 


### THE MEDICAL EXAMINER.

### PHILADELPHIA, OCT. 24, 1838.

As the season for medical studies is about commencing, we have thought that it would be agreeable to our friends at a distance to learn what are the facilities for medical instruction at Philadelphia, and in what manner the machinery of instruction actually works; for those who are conversant with such matters are fully aware that the public courses of instruction form but a portion of the whole system, and do not necessarily compose the most important part of it. We should wish to receive similar accounts of the modes which are in practice at other places. We allude, of course, to the instruction actually received by the student, whether from public or private sources. A knowledge of the various methods which are pursued, may make us somewhat acquainted with their practical defects, and thus aid us in suggesting such gradual improvements as the circumstances of the country and the profession will admit.

Persons particularly acquainted with the subject soon discover that changes of a mode of instruction, if suddenly attempted, are impracticable, and that all violent innovations annoy the pupil, perhaps induce him to content himself with an inferior course of study, and rarely persuade him to accept of the proffered improvements. The slowness of these changes or improvements in medical instruction may perhaps, in part, depend upon the reluctance felt by teachers to alter a course of instruction which, from habit, has become familiar to them; but the active efficient course is much more deeply seated. It lies with the pupils themselves, and indirectly depends on the condition of the country and the eagerness with which every one presses into the career which he has chosen. Medical instruction follows the ordinary laws of supply and demand; if a particular kind of knowledge is required, there are always a sufficient number of physicians able to communicate it; or at any rate, there is a large body of men, capable and educated, who have abundant leisure to qualify themselves for any course of instruction which the student may desire.

There is no better illustration of this matter than is offered by the history of private instruction in Philadelphia. A few years since, but a very limited number of students availed themselves of the facilities for improvement offered by physicians unconnected with the school; this number was almost exclusively confined to those who passed the whole period of their studies at Philadelphia. Now, a very large number of students attend two public courses of lectures, and remain during the intervening summer vacation; making eighteen months of continuous study. Nor is this all, a very large proportion of those who are unable to remain during the summer months, are regularly examined by competent teachers, particularly in the session immediately preceding their graduation. This kind of instruction has been gradually established and from its nature was always directly proportioned to the willingness of the students to receive it.

On the other hand, an attempt has been made, by the schools of Philadelphia, to extend their term of studies, and has succeeded but indifferently. The truth is, that those students who are desirous of diligently pursuing their studies, are completely exhausted by the fatigue of following the numerous courses of lectures during the winter season, and are but little disposed to prolong their attendance beyond the four months which have been allotted to that purpose, from long established custom. A few will amuse themselves by attending a limited number of preliminary lectures, but almost none are willing to study in good earnest for a longer period than was previously thought necessary. The necessity of a more complete course is, however, very generally felt and acknowledged, but it must be something different from the former routine; and must be something which should, as it were, arise of itself, and be adapted to the wants of the profession.

In these remarks we are not disposed to undervalue efforts of this kind; we like to see them; they are useful feelers, and must become established when they hit upon the right course. But we are anxious that such things should, in a great measure, be left to themselves, should be encouraged and be modified to meet the particular circumstances in which we are placed. It is the duty of established schools to aid in advancing the progress of medical science, and they may do this most effectually by requiring the largest amount of knowledge from those candidates for graduation, which can be expected from young men who have passed but a very short period in systematic study. This furnishes an additional stimulus, and calls attention to deficiencies, which are readily supplied as soon as they are generally admitted. These deficiences must, sooner or later, be supplied, and we believe that empirical practitioners will be the most efficient agents in enforcing a longer and more careful course of studies on the part of the regularly educated physician. It will be found, that the mere title of a graduate in an incorporated school, is not sufficient to draw a line of distinction between a physician and one who usurps the name; it must be strongly drawn by superior attainments, which are only to be purchased at much expense of time and labour. If we are right in this view, the numerous systems of quacking, which are now so rife, will confer a lasting benefit on medical science, and indirectly upon those who have attained it by a self-denying sacrifice of pleasure and indolence.

We are contented, at present, with expressing our conviction of the necessity of improvements in the course of instruction generally pursued, and of the gradual manner in which they can be brought about. We propose, in our subsequent numbers, to show what opportunities for instruction are now afforded, and we shall, perhaps, suggest such further progress as may seem practicable. We are writing, of course, for the profession, and for the pupils who are soon to form a part of it. The desire for improvement must come from them; otherwise, teachers of medicine can do but little in the matter, however honest their intentions may be.

